# Attitudes, interest and involvement of midwives in an RCT: A qualitative study with a phenomenological approach

**DOI:** 10.18332/ejm/224168

**Published:** 2026-07-10

**Authors:** Maria Panzeri, Teresa Terenghi, Sara Ornaghi, Sara E. Borrelli, Antonella Nespoli, Anna Locatelli, Simona Fumagalli

**Affiliations:** 1 School of Medicine and Surgery, University of Milano-Bicocca, Milan, Italy; 2 Department of Obstetrics, Fondazione IRCCS San Gerardo dei Tintori, Monza, Italy; 3 School of Health Sciences, The University of Nottingham, Nottingham, United Kingdom

**Keywords:** midwifery, randomized controlled trials, qualitative research, professional role, attitude of health personnel

## Abstract

**INTRODUCTION:**

In order to improve evidence-based, woman-centered care, research is an essential component of midwifery professional practice. The active involvement of clinical midwives in randomized controlled trials (RCTs) remains modest, despite its acknowledged relevance. The study aims to explore attitudes, interest, and involvement of midwives in an RCT.

**METHODS:**

A qualitative study informed by descriptive phenomenology was conducted in a second-level maternity unit in Northern Italy. Twenty-one clinical midwives participated in four focus groups prior to the implementation of an RCT evaluating a non-pharmacological intrapartum intervention. Data were audio-recorded, transcribed verbatim, and analyzed using thematic analysis supported by NVivo software.

**RESULTS:**

Four themes were identified: 1) Using research to inform practice; 2) Challenges related to RCT design features; 3) Challenges related to midwives’ involvement; and 4) Strategies to support midwives’ involvement. Midwives perceived research as essential for improving care, strengthening professional identity, and advancing midwifery within the scientific community. Evidence generation was closely linked to their commitment to woman-centered care and professional advocacy. However, RCT participation generated tensions between protocol adherence and individualized care. Increased workload, procedural complexity, and cognitive demands within busy clinical settings were described as key barriers. Facilitators included supportive leadership, the presence of a dedicated reference person, peer collaboration, preparatory and ongoing meetings, and alignment between research topics and clinical practice.

**CONCLUSIONS:**

Midwives’ engagement in RCTs is shaped not only by organizational factors but also by professional identity and relational values. Supportive research environments and structured mentorship may enhance meaningful participation and contribute to a sustainable research culture within midwifery practice.

## INTRODUCTION

In 2025, the International Confederation of Midwives (ICM) published an updated *Professional Framework for Midwifery*, outlining the core elements required for midwifery to be recognized as a profession^1^. Research is identified as a key element of professional practice, highlighting the responsibility of the profession to generate and apply scientific evidence^2^. Research conducted within midwifery and with the active participation of midwives contributes to improving practice, updating knowledge, providing evidence-based care, responding to health needs, and strengthening the scientific and professional status of midwifery^1,3,4^.

Midwifery research is essential to improving maternal and neonatal outcomes and informing clinical guidelines, policy, and service development. Studies addressing midwifery-specific interventions are particularly important to ensure that clinical practices reflect women-centered, normal, and non-pharmacological approaches to care^4-6^.

Midwives play a central role in maternity care and act as key stakeholders in the implementation of clinical research, particularly in intrapartum settings where they provide continuous care and influence both clinical decision-making and women’s participation in studies. Their engagement is therefore critical for the successful design, conduct, and implementation of randomized controlled trials (RCTs) evaluating midwifery interventions^4^.

Despite the recognized importance of midwives’ engagement in research, previous studies have reported limited participation of clinical midwives in research activities. Barriers include cultural perceptions of research, limited research training, insufficient funding, and lack of protected time within clinical workloads^7,8^.

Conversely, facilitators for research engagement include supportive leadership, collaborative research networks, organizational support, increased trust in research processes, and visibility of research outcomes within clinical settings^7,9^.

However, there is a paucity of evidence regarding the professional and personal barriers and facilitators perceived by clinical midwives when participating in research within routine clinical practice, particularly in the context of intrapartum RCTs of non-pharmacological midwifery interventions.

The aim of this study was to explore midwives’ attitudes, interest, and involvement in a randomized controlled trial of an intrapartum midwifery intervention and to identify perceived barriers and facilitators to their engagement in clinical research.

## METHODS

### Study design

A qualitative study informed by descriptive phenomenology was conducted to explore midwives’ attitudes, interests and involvement in a randomized controlled trial (RCT) of an intrapartum midwifery intervention, and to identify perceived barriers and facilitators to their engagement in clinical research. A phenomenological approach was chosen to capture midwives’ lived experiences and perceptions of participating in a clinical trial evaluating non-pharmacological intrapartum interventions. This approach is participant-oriented, allowing midwives to articulate their perspectives freely, enabling the collection of rich and in-depth data regarding professional and personal experiences of research participation.

### Research setting

The study was conducted at IRCCS San Gerardo dei Tintori Hospital, Monza, Lombardy, Northern Italy. This is a second-level maternity unit providing antenatal, intrapartum, and postnatal care, with a clinical philosophy aimed at supporting normal birth and promoting maternal and neonatal health.

### Participants and recruitment

A purposive sampling strategy was used. All midwives employed in the clinical maternity setting were eligible to participate. The only exclusion criteria were not working in clinical practice at the time of the study.

Eligible midwives received an invitation e-mail describing the study aim and procedures. Researchers were available to provide additional information and answer questions. Midwives who expressed interest provided written informed consent, including consent for audio and video recording of the focus groups, in accordance with national data protection regulations.

Twenty-one midwives were recruited and participated in the study.

To preserve anonymity in reporting, participants are identified using alphanumeric codes (P1, P2, P3, etc.) in tables and verbatim quotations. The study was reported in accordance with the Standards for Reporting Qualitative Research (SRQR) checklist^10^ (Supplementary file Material 1).

### Data collection

Data collection was undertaken between June and September 2025, using four audio and video-recorded focus groups conducted via video-call conferencing. Focus groups were chosen to facilitate shared reflection and interaction among participants. Each focus group included 5–6 participants and lasted approximately 90 minutes. Focus groups were moderated by two members of the research team (SF, MP), with a third researcher (TT) acting as observer and taking field notes.

A semi-structured topic guide was developed by the research team following a literature review on midwifery research engagement and participation in clinical trials. Topics explored experiences and perceptions of midwifery research, participation in RCTs, and perceived barriers and facilitators to research engagement in clinical practice. The topic guide was pilot-tested with two midwives not included in the study to assess clarity, relevance, and flow of questions, and refined accordingly.

### Data analysis

Focus group recordings were transcribed verbatim and anonymized. Data were analyzed using a thematic analysis informed by phenomenology, supported by NVivo software.

Two researchers (MP, TT) independently read and coded the transcripts, using an inductive-deductive approach. An initial coding framework was informed by the literature and the topic guide, while allowing new codes and themes to emerge from the data. Codes were organized into themes and subthemes, which were iteratively reviewed and refined through team discussions. Discrepancies were resolved through consensus among all authors.

Representative quotations were selected to illustrate themes and subthemes. Data collection and analysis proceeded concurrently, and recruitment ceased when thematic saturation was achieved.

Several strategies were used to enhance rigor and trustworthiness. Credibility was ensured through investigator triangulation and iterative coding. Dependability was supported by an audit trail, while confirmability was enhanced through reflexive discussions. Transferability was addressed by providing a detailed description of the study context.

Formal member checking with participants was not conducted; however, peer debriefing with academic colleagues was undertaken to enhance analytic rigor.

All researchers were midwives with clinical and academic experience. Reflexivity was maintained throughout the study through regular team discussions aimed at acknowledging how researchers’ professional backgrounds could influence data interpretation and ensuring openness to participants’ perspectives during analysis.

## RESULTS

Participant characteristics are presented in Table 1. Twenty-one midwives participated, with a mean age of 32 years and varying levels of experience. Most had previous research involvement (66%), although few had participated in RCTs (21%).

**Table 1:** Sociodemographic and professional characteristics of clinical midwives participating in a qualitative descriptive phenomenological study exploring attitudes, interest and involvement in a randomized controlled trial (RCT) of a non-pharmacological intrapartum intervention, conducted at a second-level maternity unit in Northern Italy, June–September 2025 (N=21)

*Participants*	*Age* *(years)*	*Work* *experience* *(years)*	*Workplace*	*Post-basic training*	*Previous* *involvement in* *studies*	*Study design*
P1	35	11–20	DR		No	
P2	35	11–20	DR	Master’s degree	Yes	Observational
P3	25	<5	AW/PW	Master’s degree	Yes	Observational
P4	25	<5	DR	Master’s degree	Yes	Observational
P5	27	<5	DR		No	
P6	31	5–10	AW/PW	Master’s degree	No	
P7	31	5–10	DR	Master’s degree	Yes	Observational
P8	34	5–10	AW/PW	Master’s degree/Level I Master’s degree	Yes	RCT – Topic: CMV
P9	30	5–10	DR	Level I Master’s degree	No	
P10	24	<5	AW/PW		No	
P11	29	5–10	AW/PW		Yes	Observational
P12	38	11–20	DR	Master’s degree	Yes	Not specified
P13	34	11–20	AW/PW	Master’s degree	Yes	Observational
P14	34	11–20	DR	Master’s degree	Yes	Observational
P15	26	<5	AW/PW	Master’s degree	Yes	RCT – Topic: CMV
P16	24	<5	DR	Master’s degree	Yes	Not specified
P17	28	5–10	AMB	Master’s degree/Level I Master’s degree	Yes	Observational
P18	53	>30	DR	Level I Master’s degree	Yes	Observational and RCT – Topic: aromatherapy
P19	31	5–10	DR	Level I Master’s degree	Yes	Not specified
P20	46	21–30	AMB		No	
P21	33	11–20	AMB	Level I Master’s degree	No	

DR: delivery room. AW/PW: antenatal or post-natal ward. AMB: obstetric outpatient clinic. CMV: Cytomegalovirus

The findings include four main themes: 1) Using research to inform practice; 2) Challenges related to RCT design features; 3) Challenges related to midwives’ involvement; and 4) Strategies to support midwives’ involvement. Themes and subthemes are reported in Table 2, including the number of participants and supporting quotes in which these are identified.

**Table 2:** Themes and subthemes identified through thematic analysis of four focus groups involving clinical midwives participating in a qualitative descriptive phenomenological study exploring attitudes, perceived barriers and facilitators to involvement in a randomized controlled trial (RCT) of a non-pharmacological intrapartum intervention, conducted in Northern Italy in 2025 (N=21)

*Themes and* *subthemes*	*Number of* *participants*	*Number of* *supportive* *quotes*	*Supporting quotes*
**Theme 1: Using research to inform practice**			
**Research guiding midwifery care and professional development**	20	48	*‘I believe that the role of research in our profession is somewhat the keystone for improving the care we provide, … research offers us new insights to enhance our practice.’* (P1) *‘Conducting research allows us to stay up to date with the changes in the population of women we care for and to whom we provide our services.’* (P4) *‘It is certainly something that stimulates me in my clinical decision-making … it is also nice to see how we become engaged among ourselves; it is valuable to be in that context, to talk, and from an intuition new mental processes and lines of reasoning emerge that perhaps we would not have developed before.’* (P7) *‘In my opinion, this is also somewhat a problem within midwifery … there is a great deal of empiricism.’* (P2) *‘I simply used to think of research as something that broadens and widens our perspective, even regarding what is customary, what has always been done, and what is assumed to have always been right.’* (P20) *‘If we are a scientific profession, we must position ourselves within the scientific community, just as all other professions do.’* (P17) *‘It will be an opportunity to integrate our practice … with other professionals involved in childbirth care.’* (P7)
**The value of RCT**	12	30	*‘In my opinion, the value we will have first and foremost as researchers, and therefore as a professional group, will be substantially enhanced.’* (P7) *‘The idea of conducting an RCT suggests the possibility of generating something with much stronger and more concrete evidence to support our daily practice.’* (P3) *‘Demonstrating it through a robust study allows us to disseminate the findings to a wider professional and clinical audience.’* (P4) *‘If that evidence changes clinical practice and is demonstrated by an RCT, then there are no more excuses: it must be adopted and implemented in practice.’* (P14)
**Theme 2: Challenges related to RCT design features**			
**Managing randomization processes**	10	17	*‘It is really fate that decides for you whether you end up in the intervention group or the control group.’* (P14) *‘Moreover, the ethical dimension of this must be considered within a context like ours, which involves pregnant women, newborns, and families, and therefore very sensitive situations.’* (P9) *‘It is also important to accept being in the control arm.’* (P14) *‘I find randomization challenging, not professionally, but on a personal level. Perhaps I always feel that the intervention has the potential to yield positive outcomes.’* (P2)
**Advocating for women**	8	14	*‘Moreover, I believe it is challenging to counsel women regarding participation in the study, that is, to help them understand why it is important to take part in an* *RCT*.*’* (P4) *‘Additionally, they may not fully grasp the strength of the data we obtain, as they understandably are not familiar with how scientific literature works; therefore, we need to focus on explaining this aspect to them as well.’* (P11) *‘I have observed that it is particularly helpful for a woman to preview the positions and practices that will be suggested to her … multimedia content could be incorporated into the information materials, allowing them to become familiar with these positions in advance.’* (P2) *‘So, if I am able to approach it this way, I may also be able to present the study not as “I am taking something away from you”, but as “let’s observe together what happens”.’* (P13) *‘Then, a woman may accept waiting and may feel stronger, sensing that she is capable of coping.’* (P15)
**Providing individualized care**	10	12	*‘In practice, you would decide on your own, based on your sense of personalization, what is best for each woman … here, we would be relying on a database telling us: give these three steps to one woman and something different to another.’* (P2) *‘In short, I sometimes find the concept of rigor a bit challenging, as it can conflict with the way I provide care and its personalized nature.’* (P1)
**Theme 3: Challenges related to midwives’ involvement**			
**Workload challenges**	13	15	*‘During the two postpartum hours, many times I can’t even notice first breastfeed or be with them as I would like, because I know I have to finish the bureaucracy quickly.’* (P16) *‘Moreover, there was the significant challenge that everyone had to continuously monitor the tracing, since the side effects were unknown. I remember that the entire staff was asked to make a great effort, to adhere to this approach, and to participate in a study that was part of a European project.’* (P14) *‘I do not rule out that it is very demanding at times, both regarding data collection and the time needed to clarify what has been done and to assign value to that data.’* (P7)
**Coping with unexpected outcomes**	6	7	*‘What worries me is the outcome, especially given how much one invests in an RCT, starting from a positive hypothesis and committing a lot to it. What concerns me most is that it demands so many resources and energy, and yet the result might not be what you had hoped for – this would really disappoint me.’* (P15) *‘It could even completely contradict your initial research hypothesis; therefore, you also need to be somewhat prepared to accept the final outcome.’* (P7)
**Impact of women’s knowledge and study duration on midwives’ participation**	5	11	*‘Clearly, if the study doesn’t last long, then you can manage it, but if it starts to last a long time, it could become exhausting for me.’* (P18) *‘In my opinion, it will be necessary for women to arrive informed, at a moment when they are calm, knowing what will happen, with forms already signed and prepared, and if at that point the resident performs the randomization, then that works for me.’* (P14)
**Theme 4: Strategies to support midwives’ involvement**			
**Support from study coordinators and research team**	11	14	*‘If I know that many of my colleagues are involved, I would also feel more confident in participating, because I would know with whom to share doubts or uncertainties.’* (P5) *‘Additionally, it might be a strategy to have, within the delivery room groups midwives who do occasional recaps; that is, I’m not saying a single point of reference for each shift, but one person to keep track.’* (P14) *‘Having a midwife who conducts research, who is present in the delivery room and helps us locally while we are dealing with the efforts of the shift, would, in my opinion, really help manage many doubts and also better understand any methodological issues that might arise.’* (P7)
**Interim meetings to facilitate participation**	14	22	*‘I would say that even just this meeting, simply discussing it, already highlights what the objectives are, and this happens at the beginning, before the trial has even started.’* (P7) *‘During the study, receiving feedback could help make it a little less demanding and help carry it forward.’* (P17)
**Influence of research topic**	10	17	*‘I think that if you were to propose a study on the use of rosemary oil, which has limited evidence and only marginally affects my practice, I would feel less engaged and less motivated to participate.’* (P1) *‘The fact that it is a practice I am familiar with, that I already use, and whose benefits I have directly observed in clinical practice, is certainly a positive aspect.’* (P4)
**The power of expected results**	8	12	*‘So, of course, the additional commitment is something you think about; however, if you can see its usefulness and purpose, I believe it becomes easier to maintain adherence.’* (P21) *‘However, when engaging in this type of study, I think it is important to keep that focus in mind: we are doing it for the well-being of the wider population, but also for the well-being of midwives themselves, who can improve their practice.’* (P11)
**Other facilitating factors**	10	17	*‘Another aspect that comes to mind is more practical – having data collection forms that are as streamlined as possible, focused on the study objectives, so that the time they require within my daily work remains limited.’* (P19)

### Theme 1: Using research to inform practice

Within this theme, participants described the impact of research on clinical practice. Two aspects emerged: 1) Research guiding midwifery care and professional development; and 2) The value of RCT.

*Research guiding midwifery care and professional development*

Midwives emphasized that research informs both the care provided to women and the development of the midwifery profession. First, participants highlighted the importance of research in delivering evidence-based care to women and their families. Research findings were described as a means to ‘compare’ (P2), ‘improve’ (P1), and ‘update’ (P6) clinical practice. Updating care not only responds to the needs of individual women but also aligns with the changing characteristics of the broader population. Research was also identified as a tool for providing women with the information and options they need to make an informed choice.

Secondly, research supports professional growth and critical thinking. Midwives described how engaging with research fosters reflection, critical judgment, and discussion within the team. While experience remains central to practice, research provides guidance when experience is limited, enhancing professional decision-making (P7).

Participants also noted that research challenges empiricism and traditional practice, allowing procedures to be validated scientifically rather than being based solely on habit.

Finally, research was described as a way for midwives to engage with the scientific community, collaborate with other professionals, and advance the profession.

*The value of RCT*

Midwives described a clear understanding of RCT design, highlighting its rigor and specificity. Three main benefits were identified. First, participation in RCTs elevates the professional status of midwives:

*‘In my opinion, the value we will have first and foremost as researchers,*

*And, therefore, as a professional group, will be substantially enhanced.’* (P7)

Second, RCTs provide strong, high-quality evidence that can support daily care practices:

*‘The idea of conducting an RCT suggests the possibility of generating something with much stronger and more concrete evidence to support our daily practice.’* (P3)

Third, the rigor of RCTs ensures results can be disseminated beyond a single hospital, informing guidelines and improving practice across multiple centers:

*‘If that evidence changes clinical practice and is demonstrated by an RCT, then there are no more excuses: it must be adopted and implemented in practice.’* (P14)

### Theme 2: Challenges related to RCT design features

Participants highlighted challenges associated with specific characteristics of RCTs. Three main aspects emerged: 1) Managing the randomization process; 2) Advocating for women; and 3) Providing individualized care.

*Managing the randomization process*

Midwives often described randomization as leaving the assignment to ‘destiny’:

*‘It is really fate that decides for you whether you end up in the intervention group or the control group.’* (P14)

Almost all midwives expressed fatigue and frustration with randomization for two main reasons.

First, midwifery involves ‘sensitive’ (P9) subjects – women, newborns, and families. Second, midwives reported difficulties when the proposed intervention is perceived as superior to the control, especially when validating practices already in use. Only one participant noted that the intervention may not always be the best choice for every woman. Participants also proposed strategies to cope with the challenges of randomization. These included focusing on the importance of respecting randomization to obtain robust results, enhancing communication with women, and exploring alternative ways of providing care.

*Advocating for women*

Midwives emphasized the importance of supporting women throughout all study phases – information, enrollment, randomization, and implementation. Supporting women’s advocacy involves three key steps. First, midwives need to explain the significance of participation and the potential impact of the study results:

*‘Moreover, I believe it is challenging to counsel women regarding participation in the study, that is, to help them understand why it is important to take part in an RCT.’* (P4)

Second, they must ensure women understand the intervention, sometimes using multimedia tools or demonstrations beforehand (P2). Third, effective communication should frame participation positively, emphasizing observation, fetal abilities, or shared experiences, rather than deprivation.

*Providing individualized care*

Midwives expressed difficulty in personalizing care while adhering to the strict protocol of the RCT. Personalization, based on clinical observation and the relationship between midwife and woman, often conflicted with the standardized steps required by the study design:

*‘In practice, you would decide on your own, based on your sense of personalization, what is best for each woman … here, we would be relying on a database telling us: give these three steps to one woman and something different to another.’* (P2)

This tension between individualized care and protocol rigor generated discomfort, fatigue, and self-questioning among participants.

### Theme 3: Challenges related to midwives’ involvement

This theme captures factors that can hinder midwives’ full and accurate participation in an RCT. Due to the precision and adherence required by RCT protocols, three main barriers emerged: 1) Work overload challenges; 2) Coping with unexpected outcomes; and 3) Impact of women’s knowledge and study duration on midwives’ participation.

*Work overload challenges*

Midwives highlighted that participating precisely in an RCT can increase workload in multiple ways. First, participation requires completing additional forms and documentation, which can take time away from direct care. Second, midwives described the added procedures or interventions, as well as the extra time spent reviewing and discussing the study design, as significant contributors to fatigue:

*‘I do not rule out that it is very demanding at times, both regarding data collection and the time needed to clarify what has been done and to assign value to that data.’* (P7)

These examples illustrate that work overload is not only about physical or administrative tasks but also includes cognitive and emotional effort required to follow the protocol correctly.

*Coping with unexpected outcomes*

Another barrier to participation was the fear that study results might contradict midwives’ expectations or long-held beliefs about an intervention’s effectiveness. Some midwives reported that such outcomes could generate emotional stress and fatigue, particularly given the significant resources and energy invested in the RCT. Other participants expressed readiness to accept the results, regardless of whether they aligned with their expectations.

*Impact of women’s knowledge and study duration on midwives’ participation*

Midwives also reported that participation is influenced by the duration of the study and the degree to which women are informed and enrolled. If women are already familiar with the study and ready for randomization, midwives feel participation is more manageable. This subtheme highlights how study logistics and participant readiness directly impact midwives’ ability to engage fully in research activities.

### Theme 4: Strategies to support midwives’ involvement

Participants identified several strategies to facilitate accurate, timely, and engaged participation in all phases of an RCT: 1) Support from study coordinators and research team; 2) Interim meetings to facilitate participation; 3) Influence of research topic; and 4) The power of expected results; and 5) Other facilitating factors.

*Support from study coordinators and the research team*

Midwives emphasized that having colleagues actively involved in the study, as well as a clear point of reference, enhances their confidence and engagement. Two types of support were highlighted. First, a midwife could act as a referent within each shift, helping staff manage questions or issues as they arise during routine work. Second, a midwife could serve as a dedicated contact outside the regular staff schedule, providing guidance and troubleshooting when needed. Both forms of support were considered valuable in facilitating accurate and timely participation in the RCT.

*Interim meetings to facilitate participation*

Midwives emphasized that regular meetings are crucial to support active involvement in the study. Meetings can occur at different stages and serve distinct purposes. Pre-study meetings, such as focus groups, are held before the trial begins and aim to clarify the study phases and the protocol in its entirety. Ongoing meetings during the study period were also considered important. These allow staff to stay informed about the study’s progress, discuss any challenges, and implement corrective actions when needed. Finally, one participant suggested clinical case meetings, which go beyond statistics and tables. In these sessions, midwives actively involved in the study can discuss practical strategies to overcome difficulties and share insights from direct experience.

*Influence of research topic*

Midwives highlighted that the topic under investigation strongly influences their willingness and ability to participate accurately and in a timely manner in an RCT. When the study directly impacts everyday care practices, participants reported a higher level of motivation to engage fully. In addition, practical knowledge and familiarity with the intervention were described as critical factors. Midwives reported that if they already know the procedure and have experienced its benefits firsthand, participation becomes more meaningful and manageable. Familiarity allows them not only to implement the intervention correctly but also to organize care processes, structure protocols, and train colleagues, enhancing both personal and team competence. Thus, midwives’ engagement is closely tied to the relevance of the research to their clinical practice and their prior experience with the intervention, highlighting the importance of selecting study topics that are both meaningful and applicable to everyday care.

*The power of expected results*

Midwives highlighted that keeping the potential outcomes of the study in mind strongly motivates their engagement. Awareness of the possible benefits – both for women’s health and for professional practice – helps them adhere to the protocol rigorously, even when tasks are demanding or emotionally challenging.

Participants described how focusing on the broader impact of the results – improving care for all women, enhancing midwives’ practice, and contributing to scientific knowledge – can help them persevere through the complexities and fatigue inherent in RCT participation.

This perspective underscores that anticipated results serve as a key motivational factor, linking midwives’ daily effort to meaningful, long-term improvements in care and professional development.

*Other facilitating factors*

Participants suggested several additional strategies to support effective midwife involvement in RCTs. These included one-to-one assistance, which fosters a trusting relationship with the woman; limiting non-intervention time in the control group; and ensuring that the intervention is a procedure already familiar to staff, as was the case in the trial at the reference hospital, so that no additional learning is required.

Another important factor influencing participation was the bureaucracy involved in study procedures. Midwives emphasized that data collection forms must be practical, simple, and quick to complete. Forms should focus on study objectives, be designed with input from fellow midwives, and be accessible in either paper or electronic format, according to preference and workflow. This subtheme highlights that practical considerations, workflow efficiency, and usability of study tools are critical to facilitating midwives’ engagement in research activities.

## DISCUSSION

This study explored midwives’ attitudes, interest, and involvement in RCTs, highlighting not only structural barriers and facilitators but also the relational and professional meanings attributed to research participation. Findings suggest that engagement in research is closely embedded in professional identity, professional mandate, and commitment to woman-centered care.

Midwives perceived research as essential not only for improving care but also for strengthening the profession and defining its scope of practice. Participants emphasized that research can improve midwifery care, ensure evidence-based practice, update procedures in line with the changing needs of the population, enable shared decision-making between professionals and women, stimulate critical thinking, support clinical experience when limited, foster peer-to-peer discussion among birth professionals, and contribute to the advancement of midwifery as a profession. Importantly, research was not perceived as an abstract academic exercise but as a concrete means to enhance personalized and women-centered care. In this sense, engagement in research was closely linked to advocacy: producing evidence was seen as a way to protect women’s interests and ensure that clinical practices are both safe and respectful.

These findings are consistent with the literature demonstrating that midwifery research enhances outcomes and supports woman-centered care^1,4,5^. Homer et al.^4^ emphasized that research led by midwives addresses professionals’ needs while strengthening midwifery’s scientific legitimacy. Similarly, Yeomans and Jedwab^11^ highlight that nurses and midwives recognize research as a core component of professional development and quality care delivery, reinforcing the importance of fostering research engagement across clinical settings. However, our findings extend this perspective by showing that the motivation to engage in research is not solely professional but relational. Midwives appeared motivated by the desire to offer women care that is not only evidence-based but ethically grounded and tailored to individual needs.

The alignment between participants’ perspectives and published evidence indicates that midwives, even when primarily engaged in clinical work, are aware of the importance of research in their profession, reflecting a generally positive attitude and initial enthusiasm for involvement in research.

A concept strongly emphasized by participants was evidence-based midwifery. While midwives valued integrating the best available evidence into practice, they also acknowledged the empiricism inherent in midwifery, whereby many practices are validated through experience and observation before being formally studied. De Leo et al.^12^ describe how attachment to established practices may hinder evidence translation. Participants indicated a desire to confirm experience-based methods through science, indicating a dynamic interaction between formal evidence and implicit knowledge. This result demonstrates a mature professional attitude in which research and clinical expertise are supportive rather than competitive.

Simultaneously, taking part in an RCT created a slight yet significant conflict between following the protocol and providing personalized care. Strict procedures and randomization were perceived as limiting midwives’ ability to provide tailored care. This conflict seems to arise from a deeper ethical issue – being sure that study procedures do not compromise women’s autonomy or relational continuity – rather than methodological doubt. The difficulty of participating in RCTs may therefore be more about integrating standardized methodologies with the relational ethos of midwifery practice than it is about technical complexity.

Regarding involvement in research, participants identified increased workload and competing clinical priorities as significant barriers. As reported in previous studies^5,11,13-15^, the lack of protecting time required midwives to incorporate research-related tasks into already demanding clinical shifts. Our results, however, suggest that the strain went beyond time constraints. The cognitive complexity of study participation in clinical settings is demonstrated by the participants’ descriptions of the effort required to maintain protocol adherence while providing personalized care. Although workload has been extensively documented, more attention should be given to the mental and practical challenges of incorporating research into daily care.

Supporting women’s advocacy, understanding the value of sharing research findings, and providing continuous guidance and support during the study were all identified as facilitators in this study. These results are supported by the literature, which emphasizes the significance of more confidence, visibility, and adequate support for professionals conducting research^7,16-21^. Fostering women’s advocacy enables informed and shared decision-making, which is only feasible when midwives are confident in their ability to provide evidence-based information. Midwives are motivated to participate when they can see the wider influence of their work through visibility and results sharing.

Our findings align with literature emphasizing mentorship, leadership and organizational support as pivotal to research engagement, providing guidance and ensuring adherence to the protocol^11,12,18,19^. These findings are also supported by Henshall et al.^21^ who stress the importance of structured mentorship, leadership, and organizational support in promoting research engagement among midwives. However, our findings point to a further layer: networks of support reduce the anxiety associated with handling difficult tasks through more than just facilitating logistical adherence. Midwives’ initial fear was replaced by active and meaningful participation when they felt supported and appreciated. In this way, it seems that relational safety in the workplace is just as important to research involvement as structural facilitators.

Overall, our results show that professional identity, relational ethics, and organizational setting interact to influence midwives’ involvement in RCTs. Therefore, strategies that acknowledge the relational nature of the profession and promote supportive, collaborative workplaces are just as important for strengthening the research culture in midwifery as training and protected time.

### Strength and limitations

The recruiting of midwives who were going to participate in an RCT with immediate implications for clinical practice is one of the study’s main strengths. This scheduling increased the findings’ ecological significance by enabling participants to consider their involvement in the research in light of an impending and tangible professional experience. The findings were enhanced by the participants’ varied backgrounds in terms of age, education, experience, and previous exposure to research. Transferability might be constrained by the single-site design, though. Furthermore, self-selection bias may have been introduced by the voluntary purposive sampling, which might have overrepresented midwives who were more favorable to research.

## CONCLUSIONS

This study highlights that midwives’ engagement in randomized controlled trials is shaped by both organizational factors and professional identity, particularly within a woman-centered model of care. While RCTs are valued for their potential to strengthen evidence-based practice and professional recognition, participation may generate tensions between protocol adherence and personalized care, alongside practical and cognitive demands in clinical settings.

Facilitating meaningful engagement requires supportive organizational structures, including adequate coordination, opportunities for communication within research teams, and alignment between research topics and clinical practice. However, our findings also suggest that effective participation depends on relational and professional dimensions, highlighting the importance of fostering supportive and collaborative research environments within maternity care settings.

Further research should explore how ethical and relational tensions between standardized research designs and individualized care can be better addressed, and how institutional context influences midwives’ sustained engagement in research.

## Supplementary Material



## Data Availability

The data supporting this research are available from the authors on reasonable request.
