# Supplementary material for: Attitudes, interest and involvement of midwives in an RCT: A qualitative study with a phenomenological approach
Source: Eur J Midwifery. 2026 Jul 10;10:10.18332/ejm/224168. doi: 10.18332/ejm/224168 (PMC13353166; doi:10.18332/ejm/224168)
Supplement: Supplementary file 1 [file EJM-10-30-s1.pdf]

EUROPEAN JOURNAL
OF MIDWIFERY

Supplementary file

© 2026 Panzeri M. et al.

DOI: 10.18332/ejm/224168

The content has been provided by the author(s) and has not been reviewed, verified, or endorsed by European Publishing. It may not have undergone peer review. The views, opinions, and recommendations expressed are solely those of the author(s) and do not necessarily reflect the position of European Publishing. European Publishing accepts no responsibility or liability for any consequences arising from the use of, or reliance on, this content.

Supplementary material 1: Standards for Reporting Qualitative Research (SRQR) checklist[10] completed for the present study, indicating where each reporting item is addressed within the manuscript.

#	Item	Description	Reported on page n°
1	Title	Concise description of the nature and topic of the study	1
2	Abstract	Summary of key elements (background, purpose, methods, results, conclusions)	1
3	Problem formulation	Description and significance of the problem/phenomenon studied	2
4	Purpose or research question	Study purpose and specific objectives or questions	2
5	Qualitative approach and research paradigm	Approach (e.g., IPA) and underlying paradigm	2-3
6	Researcher characteristics and reflexivity	Researchers' background, relationship with participants, assumptions	3-4
7	Context	Setting/site and relevant contextual factors	3
8	Sampling strategy	How and why participants/documents/events were selected	3
9	Ethical issues pertaining to human subjects	Ethics approval, consent, confidentiality	4
10	Data collection methods	Type of data collection and procedures	3

11	Data collection instruments and technologies	Interview guides, tools, software used	3-4
12	Units of study	Number and characteristics of participants	4
13	Data processing	Transcription, data management, anonymization	3-4
14	Data analysis	Analytical process (coding, themes, etc.)	3-4
15	Techniques to enhance trustworthiness	Credibility, triangulation, reflexivity, etc.	3-4
16	Synthesis and interpretation	Main findings/themes	4
17	Links to empirical data	Use of quotes or examples to support findings	4-9
18	Integration with prior work	Comparison with existing literature	9-11
19	Implications	Practical, theoretical, or policy implications	9-12
20	Limitations	Study limitations	11
21	Conflicts of interest	Disclosure	11
22	Funding	Sources of funding and role of funders	11-12
